# Developmental Axon Degeneration Requires TRPV1-Dependent Ca^2+^ Influx

**DOI:** 10.1523/ENEURO.0019-19.2019

**Published:** 2019-02-27

**Authors:** Aaron D. Johnstone, Andrés de Léon, Nicolás Unsain, Julien Gibon, Philip A. Barker

**Affiliations:** 1 Department of Neurology and Neurosurgery, Montreal Neurological Institute, McGill University, Montreal, Quebec H3A 2B4, Canada; 2 Department of Biology, University of British Columbia Okanagan, Kelowna, British Columbia V1V 1V7, Canada; 3 Instituto de Investigación Médica Mercedes y Martín Ferreyra, INIMEC-Consejo Nacional de Investigaciones Científicas y Técnicas (CONICET), Universidad Nacional de Córdoba, Córdoba 5016, Argentina

**Keywords:** calcium, neurodegeneration, NGF, pruning, TrkA, TRPV1

## Abstract

Development of the nervous system relies on a balance between axon and dendrite growth and subsequent pruning and degeneration. The developmental degeneration of dorsal root ganglion (DRG) sensory axons has been well studied in part because it can be readily modeled by removing the trophic support by nerve growth factor (NGF) *in vitro*. We have recently reported that axonal fragmentation induced by NGF withdrawal is dependent on Ca^2+^, and here, we address the mechanism of Ca^2+^ entry required for developmental axon degeneration of mouse embryonic DRG neurons. Our results show that the transient receptor potential vanilloid family member 1 (TRPV1) cation channel plays a critical role mediating Ca^2+^ influx in DRG axons withdrawn from NGF. We further demonstrate that TRPV1 activation is dependent on reactive oxygen species (ROS) generation that is driven through protein kinase C (PKC) and NADPH oxidase (NOX)-dependent pathways that become active upon NGF withdrawal. These findings demonstrate novel mechanistic links between NGF deprivation, PKC activation, ROS generation, and TRPV1-dependent Ca^2+^ influx in sensory axon degeneration.

## Visual Abstract

**Figure:**
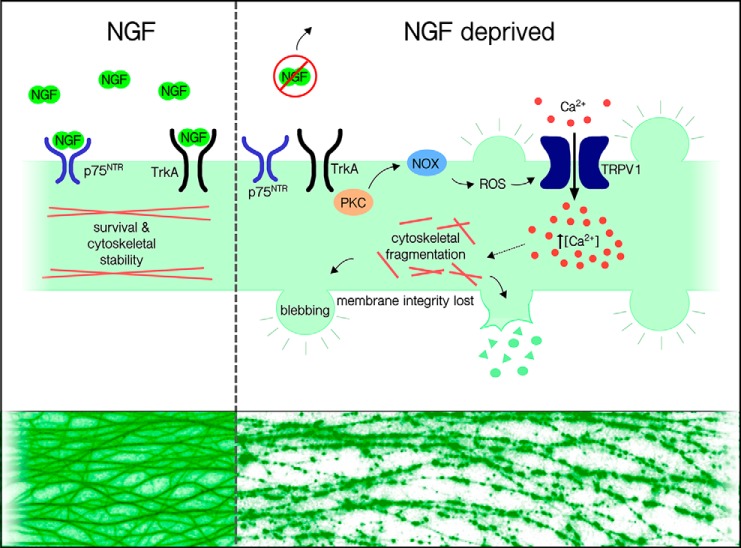

## Significance Statement

Neurons are equipped with the genetic means to degenerate, and a subset of peripheral neurons normally degenerate during embryonic development to establish a mature pattern. This beneficial neurodegeneration is regulated by signaling pathways that are only partially understood, yet share components with pathways that mediate pathologic degeneration of crucial neural structures during adult diseases such as Alzheimer’s and Parkinson’s. Here, we identify transient receptor potential vanilloid family member 1 (TRPV1) as a key regulator of Ca^2+^ entry into axoplasm that is required for developmental degeneration modeled by nerve growth factor (NGF) withdrawal from sensory neurons of the dorsal root ganglion (DRG) *in vitro.* Crucially, we report that the TRPV1-mediated Ca^2+^ flux is prompted by a signaling axis comprised of protein kinase C (PKC)-dependent NADPH oxidase (NOX) complex activation and reactive oxygen species (ROS) generation upstream of TRPV1.

## Introduction

Axons normally degenerate during embryonic development to refine the nervous system into its mature pattern (Patel et al., 2000; Schuldiner and Yaron, 2015). In the dorsal root ganglia (DRG), greater numbers of sensory neurons are generated than will persist, and surviving neurons are those that arrive at their targets and receive adequate neurotrophic support from a limited pool secreted from their targets (Barde, 1989; Vogelbaum et al., 1998; Saxena and Caroni, 2007). Genetically-encoded components of developmentally-required signaling pathways that mediate the removal of neurites [including tumor necrosis factor receptors (TNFRs), MAP kinases, Bax, and caspases] also underlie neurodegenerative diseases such as Alzheimer’s, Parkinson’s and amyotrophic lateral sclerosis (ALS) when they are dysregulated in adulthood (Kirkland and Franklin, 2003; Fischer and Glass, 2007; Saxena and Caroni, 2007; Vickers et al., 2009; Tait and Green, 2010; Kanaan et al., 2013).

We recently reported that sensory axons are rescued from developmental degeneration by Ca^2+^ chelation *in vitro* (Johnstone et al., 2018). Intriguingly, nerve terminals that innervate the skin can be locally ablated in the clinic by activation of Ca^2+^ influx mediated by the cation channel transient receptor potential vanilloid family member 1 (TRPV1); topical application of the TRPV1 agonist capsaicin is used to alleviate chronic pain and itch in humans (Jancso et al., 1985; Gibbons et al., 2010; Chiang et al., 2015; Şavk, 2016). Since activation of TRPV1 can trigger degeneration in sensory neurons (Jancso et al., 1985; Sann et al., 1995; Wang et al., 2001; Gibbons et al., 2010; Chiang et al., 2015), and because we found that Ca^2+^ is required for developmental degeneration (Johnstone et al., 2018), here we have explored the possibility that TRPV1 is required for developmental degeneration of sensory axons.

TRPV1 was identified through expression cloning designed to find the gene product that mediates Ca^2+^ influxes in response to capsaicin (Julius et al., 1997). In the intervening 20 years, TRPV1 has been confirmed to be activated and/or sensitized by heat, protons, reactive oxygen species (ROS), by the endogenous compounds *N*-arachidonoyl dopamine (NADA) and anandamide, by direct oxidation and by an array of noxious pest-defense compounds produced by invertebrates and plants (Hakim et al., 2015; Fenwick et al., 2017; Geron et al., 2017; Grabiec and Dehghani, 2017).

ROS were initially considered a toxic consequence of aerobic respiration, but a large number of studies published in the past two decades have firmly established ROS as second messengers that regulate several signaling cascades (Li et al., 2006, 2009; Bedard and Krause, 2007; Ibi et al., 2008; Pal et al., 2013). NADPH oxidase (NOX) complexes are membrane-associated protein complexes that reduce NADPH to generate superoxide from molecular oxygen. The canonical NOX complexes are activated by protein kinase C (PKC), which targets the p47phox subunit for phosphorylation. NOX complexes are major sources of ROS species that regulate survival, plasticity and degeneration (Cox et al., 1985; Maher and Schubert, 2000; Li et al., 2006, 2009; Ibi et al., 2008; Petry et al., 2010; Reczek and Chandel, 2015).

In this study, we show that axons deprived of nerve growth factor (NGF) display Ca^2+^ influx before fragmentation. This Ca^2+^ influx was prevented by TRPV1 inhibition or TRPV1 genetic knock-out. We hypothesized that ROS are required for TRPV1 activation and the resulting Ca^2+^ influx, and in support of this, we found that ROS scavengers and NOX complex inhibitors rescue axons from Ca^2+^ influx and fragmentation. PKC activity was necessary for the NGF deprivation-induced Ca^2+^ influx and degeneration that we observed, and direct activation of PKC was sufficient to drive robust Ca^2+^ influx into axons. We reasoned that PKC, NOX complexes, ROS, TRPV1, and Ca^2+^ comprise a signaling axis in these axons and consistent with this, found that PKC functions upstream of NOX, and ROS to mediate TRPV1-dependent Ca^2+^ influx. Taken together, these results show that NGF deprivation induces a Ca^2+^ influx via TRPV1 downstream of PKC and NOX complex-derived ROS during developmental axon degeneration.

## Materials and Methods

### Dissection, culturing, and NGF deprivation of DRG explants

DRG explants were dissected from pregnant CD1 mice with litters of embryonic day E13.5 embryos (Charles River). Explants were seeded on six-well plastic cell culture plates (Greiner) or four-well glass-bottom imaging dishes (CellVis) coated in a three-step process with 1 mg/ml poly-D-lysine (Sigma-Aldrich), 10 μg/ml laminin-entactin complex (Corning), and PurCol bovine collagen 0.1 mg/ml (Advanced Biomatrix). Explants were cultured in neurobasal medium (Invitrogen) supplemented with 2% B_27_ serum-free supplement (Invitrogen), 1% L-glutamine (Wisent), 1% penicillin/streptomycin (Wisent), and 10 μM 5-fluoro-2’-deoxyuridine (FDU; Sigma-Aldrich) with 12.5 ng/ml NGF (CedarLane). NGF deprivation was achieved using fresh media as described above but lacking NGF and containing 2.8 μg/ml rabbit anti-NGF antibody (produced in-house). A full description of the anti-NGF antibody (raised against 2.5s NGF), its specificity and its biological validation in survival and cell-surface binding assays has been published (Murphy et al., 1993). All experimental procedures were approved by the Montreal Neurologic Institute Animal Care Committee and University of British Columbia animal care committees and were in compliance with Canadian Council on Animal Care guidelines.

### Fixation, immunostaining, and imaging

DRG cultures were fixed in 4% paraformaldehyde in PBS for 15 min at room temperature and permeabilized and blocked for immunostaining in TBS-T, 5% skim milk, and 0.3% Triton X-100 for 15 min at room temperature. Immunostaining was performed in TBS-T with 5% skim milk and 0.3% Triton X-100 with mouse anti-β-III tubulin (Millipore; 1:10,000) primary antibody and anti-mouse secondary antibody conjugated to Alexa Fluor 488 (ThermoFisher; 1:5000). Cultures were imaged at 5× magnification using a Zeiss ObserverZ.1 inverted epifluorescence microscope with an automated, motorized stage. Images were stitched automatically with Zen 2 software from Zeiss to produce a master image of all explants on the entire six-well plate. From this master image, quarter-DRG fields were cropped using NIH ImageJ (FIJI build) to create an image set for quantification as recently described in detail by our group (Johnstone et al., 2018).

### Quantification of axon degeneration

Axon degeneration was quantified by implementing the R script Axoquant2.0 recently described in detail and made freely available by our group (Johnstone et al., 2018). For plotting and statistical analysis, each point represents the mean value of measurements from the complement of DRG cultured from a single embryo.

### Ca^2+^ chelation

After 60h of growth in NGF, cultures were either maintained in NGF or were deprived of NGF and exposed to anti-NGF antibody (2.8 µg/ml, produced in-house) in the presence of EDTA 6 mM (Sigma-Aldrich) for the final 12 h or for the entire 24 h before fixation with paraformaldehyde 4% in PBS, stained as described above and imaged for quantification.

### Antioxidant preparation

N-acetylcysteine (NAC; Sigma-Aldrich) was prepared at 20 mM in neurobasal media and pH adjusted to 7.4.

### Pharmacological PKC, NOX complex, and TRPV1 inhibitors

PKC inhibitors Gö6976 and Gö6983, NOX complex inhibitors diphenyliodonium (DPI), apocynin and VAS2870, and TRPV1 inhibitor capsazepine (all obtained from Tocris) were prepared in dimethylsulphoxide (DMSO) and delivered to cultures at 10 μM. DMSO did not exceed 0.1% in final culture in any case.

### Generation of mixed-genotype TrpV1 embryo litters

C57BL6 mice carrying the TRPV1^tm1Jul^ (targeted mutation 1, David Julius) knock-out allele in homozygosity were obtained from The Jackson Laboratory and crossed with wild-type C57BL6 mice to generate TRPV1^-/+^ animals, and these heterozygotes were bred in timed pregnancies to produce mixed-genotype litters (confirmed by PCR; for primer squences, see Table 1) of wild-type and *TRPV1* ^-/-^ E13.5 embryos for DRG isolation.

**Table 1.** Materials list

Reagent	Source	Identifier
Antibodies		
Mouse anti-tubulin beta III	EMD Millipore	MAB5564
Goat anti-mouse Alexa Fluor 488	Jackson ImmunoResearch	115-545-003
Goat anti-mouse HRP	Jackson ImmunoResearch	115-053-146
Mice		
CD1	Charles River	IMSR catalog #CRL:22, RRID:IMSR_CRL:22
B6.129X1-*Trpv1^tm1Jul^*/J	The Jackson Laboratories	3834761, RRID:MGI:3834761
Software		
Prism 6	GraphPad	RRID:SCR_002798
Inkscape 0.91	The Inkscape Project	RRID:SCR_014479
FIJI	NIH	RRID:SCR_002285
Axoquant2.0	Johnstone et al. (2018)	http://www.github.com/BarkerLabUBC
RStudio 1.1.442	RStudio	RRID:SCR_000432
ZEN 2	Zeiss	RRID:SCR_013672
LAS X	Leica	RRID:SCR_013673
Culture reagents		
Neurobasal	Gibco	21103049
B-27	Gibco	17504-044
Penicillin/streptomycin	Gibco	15140122
GlutaMax	Gibco	35050-061
NGF	CedarLane	CLMCNET-001
Poly-D-lysine	Sigma	P6407-5MG
Laminin/entactin	Corning	08-774-555
Collagen	PurCol	5005-100ML
Oligonucleotides		
For *TrpV1* genotyping (wild-type forward) TGGCTCATATTTGCCTTCAG	Invitrogen	19922
For *TrpV1* genotyping (mutant forward) TAAAGCGCATGCTCCAGACT	Invitrogen	oIMR1627
For *TrpV1* genotyping (in-common reverse) CAGCCCTAGGAGTTGATGGA	Invitrogen	19923

### End-point Ca^2+^ imaging with Fluo-4

Thirty minutes before imaging, cultures were incubated with 5 μM Fluo-4 (Invitrogen) in DMSO (Sigma, final concentration in media did not exceed 0.1%) for 15 min at 37°C and then allowed to equilibrate in fresh, room-temperature HBSS (Wisent) supplemented with 2 mM CaCl_2_ for another 15 min. Imaging was performed in fresh HBSS with 2 mM CaCl_2_ with a Leica DMi8 confocal microscope and LAS X software with a 488-nm laser in 0.3-μm z-increments with 63× objective to capture at least two fields of axons per ganglia. Background was corrected from images of sum-z-stacks by averaging the mean pixel intensities within four background regions and subtracting this value from each pixel in the image using ImageJ (FIJI build, NIH). The 2D area occupied by axons in each image was then measured using a binary mask of all axons, and the mean pixel intensity value for each image was divided by the area occupied by axons to provide a measure of Fluo-4 fluorescence intensity per unit axon area. Field images of DRG axons from the same embryo were averaged to produce the embryo mean value. In each experiment, measurements were standardized to the NGF control value of 1.0 and the size of the treatment values expressed as fold-change from NGF control.

### Live Ca^2+^ imaging with Fluo-4

Thirty minutes before imaging, cultures were incubated with 5 μM Fluo-4 (Invitrogen) in DMSO (Sigma, final concentration in media did not exceed 0.1%) for 15 min at 37°C and then allowed to equilibrate at room temperature, then washed with fresh HBSS (Wisent) supplemented with 2 mM CaCl_2_ (Fisher BioReagents) for 15 min. Fields of >100 axons each were acquired at 40× at a rate of one frame every 5 s on a Zeiss ObserverZ.1 inverted epifluorescence microscope controlled by ZEN2 software with an atmosphere-controlled incubation chamber (Pecon) and 470-nm Colibri LED light source (Zeiss). Before injection of stimulus [phorbol 12-myristate 13-acetate (PMA), 100 nM] baseline fluorescence was recorded for 1 min (12 frames). Background was corrected from each frame individually in each movie by averaging the pixel intensity value of four background regions and subtracting this value from the mean pixel intensity of the uncorrected frame (NIH ImageJ, FIJI build). Fluo-4 responses were standardized for each run as fold-change from the initial frame (set to 1.0).

### Live Ca^2+^ imaging with GCaMP6f

At time of seeding, DRG cultures were infected with HSV-hEF1-GCaMP6f (Massachusetts Institute of Technology Viral Core Facility) followed by 24 h to allow for neurite growth and GCaMP6f expression. Images of NGF-supplied or deprived axons were acquired at a rate of one every 10 min on a Zeiss ObserverZ.1 inverted epifluorescence microscope. Multiple field positions were imaged with an automated stage and atmosphere-controlled incubation chamber (Pecon) controlled by ZEN2 software, and 40× objective using a 470-nm Colibri LED light source (Zeiss). Axons were cropped from these movies and background was corrected on each frame using ImageJ (FIJI build, NIH) by averaging background regions immediately adjacent to either side of the axon and subtracting this value from each pixel of the uncorrected frame. To standardize fluorescence intensity to the time of morphologic degeneration, the frame where axon collapse accompanied by membrane spheroids was observed was considered time = 0, and time-course values were then standardized to the corrected intensity value of the frame acquired 180 min prior and expressed as fold-change from 1.0.

### Experimental design and statistical analysis

Data were plotted and analyzed with Prism 6 (GraphPad). One-factor ANOVA with Dunnett’s *post hoc* comparisons were used to analyze the effects of capsazepine, NAC, VAS2870, Gö6976, and Gö6983 on Fluo-4 intensity standardized to the mean NGF control value, the effect of capsazepine on axon density after NGF deprivation versus the NGF-deprived control, and the effect of NGF deprivation on GCaMP6f response (RM in the “time” factor). Two-factor ANOVA was used to test the effect of EDTA on axon density (RM in the “distance from soma” factor and Dunnett’s *post hoc* comparisons with NGF-deprived control) and the effect of NAC, VAS2870, Gö6976, and Gö6983 on axon density (Tukey’s *post hoc* comparisons and RM in the “distance from soma” factor). Two-factor ANOVA was also used to analyze the effect of TRPV1 knock-out on axon density after NGF deprivation and to assess the effect of capsazepine on maximum Fluo-4 response to PMA (Tukey’s *post hoc* comparisons made in each case). A two-way ANOVA (RM in the time factor) with Sidak’s multiple comparisons was performed on data collected during time-course imaging of the Fluo-4 response to PMA in wild-type and *TrpV1*-null axons. Unpaired, two-tailed *t* tests were used to test the significance of the Fluo-4 response to PMA and NGF deprivation and to test the effect of TrpV1 knock-out on PMA responses. Plotted values in each case represent the mean of a single embryo, and the number of embryos *n* in each experiment and condition is described in corresponding figure legends. Full statistical results are available on request.

## Results

### Ca^2+^ influx is required for axon degeneration

**Figure 1.**
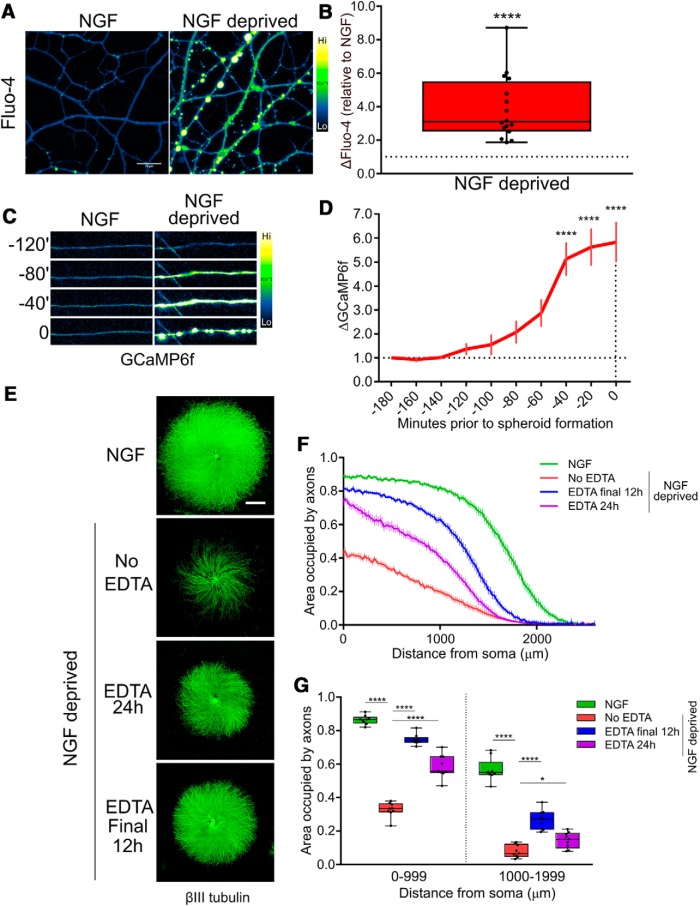
Ca^2+^ is required for developmental degeneration *in vitro*. ***A***, Cultures of DRG explants were either maintained in NGF or deprived of NGF for 15 h before loading with Ca^2+^ sensor Fluo-4. ***B***, Axons deprived of NGF displayed a significantly elevated axonal Ca^2+^ concentration (data standardized to NGF, *n* = 16, compiled from NGF and deprived controls; analyzed by unpaired two-tailed *t* test and indicated are median, min/max, and 25/75%). ***C***, Axoplasmic Ca^2+^ influx reported by GCaMP6f occurred proximal to the time of morphologic degeneration of the axon. ***D***, Axoplasmic Ca^2+^ increase was significantly elevated by 40 min before membrane spheroid formation but not earlier as compared to intensity 180 min before spheroids (indicated are mean and SEM; one-factor ANOVA and Dunnett’s *post hoc*). ***E***, βIII-tubulin staining of DRG explants treated with EDTA after 12 h of NGF deprivation or for the entire 24-h deprivation phase. Consistent with a late role for Ca^2+^ in axon degeneration, axons were significantly rescued from cytoskeletal fragmentation even when Ca^2+^ dynamics were left unmodulated by chelation during the first 12 h of NGF deprivation. ***F***, Axoquant2.0 output curves are shown with mean and SEM (*n* = 9 embryos from three pooled litters). ***G***, Axon density within 1000-μm bins were analyzed by two-factor ANOVA and Dunnett’s *post hoc* comparison and plotted with median, min/max, and 25/75%; *p < 0.05, ****p < 0.0001.

We previously showed that chelation of extracellular Ca^2+^ by EGTA rescues axons from trophic withdrawal-induced degeneration (Johnstone et al., 2018). To confirm that NGF deprivation induces an increase in axoplasmic Ca^2+^, DRG axons were withdrawn from NGF and examined by Ca^2+^ imaging using the dark-to-bright Ca^2+^-responsive dye Fluo-4. Figure 1*A*,*B* shows that axoplasmic Ca^2+^ is significantly increased at 15 h of NGF withdrawal. To understand the kinetics of the Ca^2+^ increase relative to the timing of membrane spheroid formation and frank degeneration, axons were infected with herpes simplex virus (HSV) harboring the genetically-encoded Ca^2+^ sensor GCaMP6f and live-imaged after NGF deprivation to record the timing of Ca^2+^ rise (Fig. 1*C*). Because the degeneration of individual axons is not perfectly synchronized, videos were standardized to the frame in which axons acquired membrane spheroids; this approach revealed that an increase in the GCaMP6f signal is initiated approximately 2 h prior to morphologic degeneration and peaks in the last 40 min before frank axon breakdown (Fig. 1*D*). Thus, the Ca^2+^ influx occurs before the emergence of gross morphologic changes.

Since NGF deprivation induced a significant axoplasmic Ca^2+^ influx proximal to membrane spheroid formation, and we recently reported that Ca^2+^ chelation rescues axons from degeneration (Johnstone et al., 2018), we sought to clarify whether Ca^2+^ signaling is required during the early phase after NGF deprivation to induce degeneration, or whether Ca^2+^ is only required as a late event. DRG neurons were grown in NGF and then either maintained in NGF, deprived of NGF or deprived of NGF in the presence of EDTA added at the beginning of the deprivation phase (EDTA 24 h) or only after the first 12 h of deprivation (Fig. 1*E*). Axon density versus distance was quantified using Axoquant2.0 (Fig. 1*F*). Axons were rescued from degeneration when EDTA was added for the full 24 h of NGF withdrawal or when added for the final 12 h (Fig. 1*G*). Intriguingly, axons were more robustly protected when EDTA was added only for the final 12 h of NGF deprivation, versus deprived axons continuously supplied with EDTA for the entire 24-h deprivation period, likely indicative of the important role for Ca^2+^ in outgrowth and survival pathways. We conclude that Ca^2+^ influx is a late event in axonal degeneration induced by NGF withdrawal.

### TRPV1 mediates developmental degeneration

**Figure 2.**
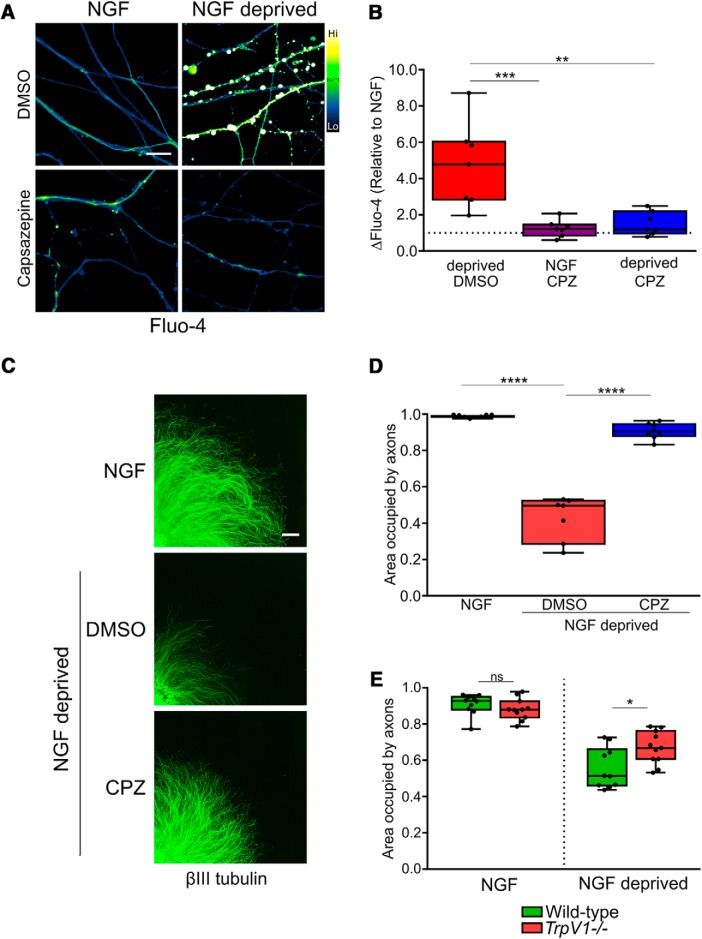
TRPV1 mediates Ca^2+^ flux and cytoskeletal fragmentation during trophic factor deprivation. ***A***, TRPV1 inhibition by capsazepine rescued axons from Ca^2+^ influx. Axons were loaded with Fluo-4 after the indicated treatments and imaged by confocal microscopy. ***A***, Fifteen hours of NGF deprivation induced robust activation of Ca^2+^ sensor Fluo-4 in axons, but co-application of 10 μM capsazepine (TRPV1 inhibitor, CPZ) ablated the response as compared to deprived controls (quantified in ***B***; *n* = 7 pooled experiments, one-factor ANOVA and Dunnett’s *post hoc* comparison to the deprived condition). ***C***, ***D***, NGF deprivation for 24 h resulted in a significant loss of tubulin-stained axons, but addition of 10 μM CPZ after 12 h of trophic withdrawal for the final 12 h resulted in a rescue of axon density to a level not significantly different from healthy controls (*n* = 8 embryos in NGF and capsazepine conditions, *n* = 7 in DMSO; one-factor ANOVA and Dunnett’s *post hoc* comparison were performed on axon density in a bin between 1000 and 1999 μm from soma. ***E***, TRPV1 knock-out rescued axons from cytoskeletal degeneration; neurons cultured from mixed-genotype litters were deprived of NGF for 24 h, after which TRPV1-null axons were significantly more dense than axons in cultures derived from wild-type animals. Axon density between 1000 and 1999 μm from soma was compared by two-factor ANOVA and Tukey’s *post hoc* comparison. Indicated are median, min/max, and 25/75% in each panel; *p < 0.05, **p < 0.01, ***p < 0.001, ****p < 0.0001.

Capsaicin-induced activation of TRPV1 can induce Ca^2+^-dependent degeneration of sensory nerve fibers (Jancso et al., 1985; Gibbons et al., 2010; Chiang et al., 2015; Şavk, 2016), but to date, the role for TRPV1 in developmental degeneration has not been explored. To address this, we deprived DRG sensory neurons of NGF in the presence or absence of TRPV1 antagonist capsazepine and measured axonal Ca^2+^ levels using Fluo-4. Figure 2*A* shows that the increase in axonal Ca^2+^ concentration that normally occurs following NGF deprivation was lost in the presence of the TRPV1 inhibitor (quantified in Fig. 2*B*). Further, the fragmentation of the axonal tubulin cytoskeleton that is normally observed after 24 h of NGF deprivation was substantially reduced in axons treated with capsazepine (Fig. 2*C*,*D*) as well as in TRPV1^-/-^ DRG axons (Fig. 2*E*). We conclude that TRPV1-dependent Ca^2+^ entry plays an important role in developmental sensory axon degeneration.

### ROS generated by NOX complexes are required for degeneration

**Figure 3.**
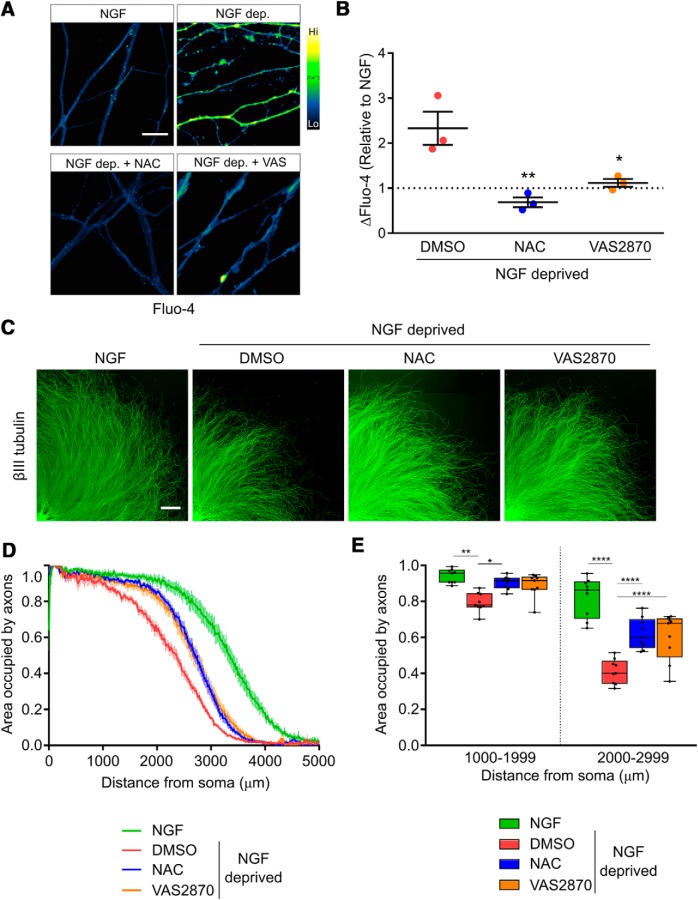
ROS derived from NOX complexes activate Ca^2+^ flux and axonal degeneration *in vitro*. ***A***, DRG explants cultured in NGF were either maintained in NGF or withdrawn from trophic factor for 15 h before staining with Fluo-4 and imaged by confocal microscopy. ***B***, Antioxidant NAC (20 mM) or NOX complex inhibition using VAS2870 (10 μM) significantly impaired axonal Ca^2+^ influx induced by trophic factor withdrawal (*n* = 3 pooled experiments standardized to the NGF condition, one-factor ANOVA and Dunnett’s *post hoc* comparison; mean and SEM are indicated). ***C–E***, Antioxidant or NOX complex inhibition significantly rescued DRG axonal cytoskeleton (visualized with βIII-tubulin immunostaining) when added after 12 h of NGF deprivation for the final 12 h. ***D***, Axoquant2.0 axon density output curves with mean and SEM. ***E***, Axon density was analyzed within 1000-μm bins using two-factor ANOVA and Tukey’s *post hoc* analysis. Median, min/max, and 25/75% are indicated; *p < 0.05, **p < 0.01, ****p < 0.0001.

ROS are important physiologic regulators of TRPV1 (Salazar et al., 2008; Ding et al., 2016; Hargreaves and Ruparel, 2016; Ogawa et al., 2016). To explore the possibility that ROS are generated following NGF deprivation, we first asked whether the rise in axonal Ca^2+^ that occurs after NGF deprivation is blocked in the presence of NAC, a potent antioxidant. Figure 3*A*,*B* shows that NAC completely blocked the rise in axonal Ca^2+^ influx that normally occurs 15 h after NGF deprivation.

Under normal physiologic circumstances, a major source of intracellular ROS are NOX complexes (Li et al., 2006; Cao et al., 2007; Ibi et al., 2008; Kallenborn-Gerhardt et al., 2014; Spencer and Engelhardt, 2014). To determine if NOX complex activity contributes to the axonal Ca^2+^ influx that occurs in DRG sensory axons after NGF withdrawal, we exposed axons to NOX complex inhibitor VAS2870. Figure 3*A*,*B* shows that VAS2870 blocks the Ca^2+^ influx that normally occurs 15 h after NGF deprivation. Both NAC and VAS2870 rescue axons from degeneration following NGF withdrawal (Fig. 3*C–E*). Axon protection was also conferred by two additional NOX complex inhibitors besides VAS2870, apocynin and DPI (data not shown). Therefore, we conclude that NOX-dependent ROS generation plays a critical role in developmental axon degeneration.

**Figure 4.**
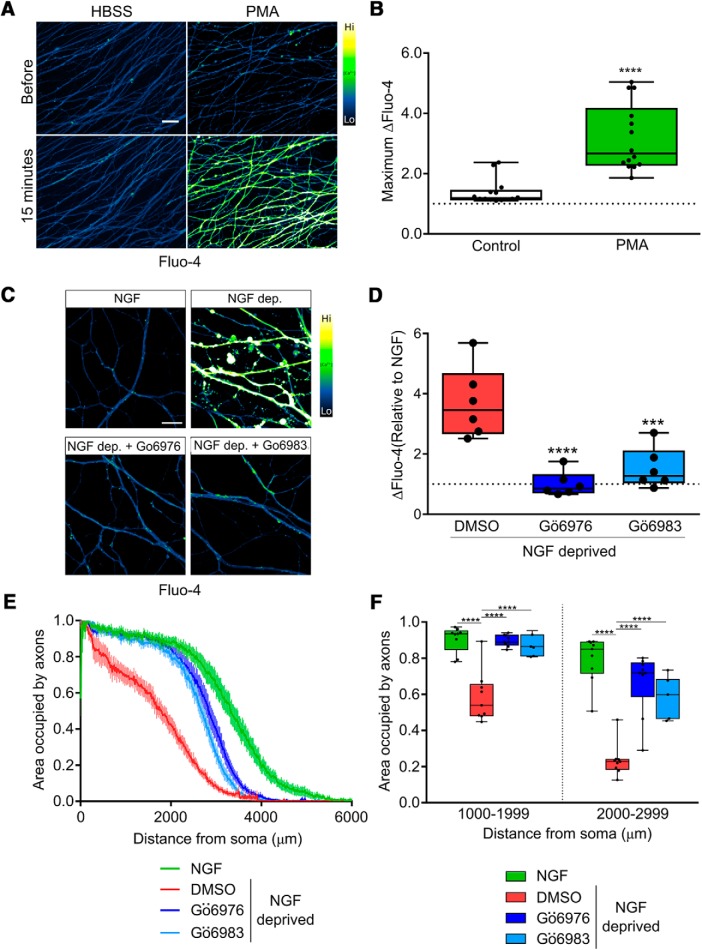
PKC mediates Ca^2+^ flux and axonal degeneration *in vitro*. ***A***, Representative micrographs of DRG axons loaded with Fluo-4 and treated with PKC activator PMA (100 nM). ***B***, Direct PKC stimulation by PMA is sufficient to activate axonal Ca^2+^ influx (*n* = 14 embryos pooled from PMA and buffer controls and compared using an unpaired, two-tailed *t* test.) ***C***, ***D***, Ca^2+^ influx activated by 15 h of NGF deprivation is significantly rescued when PKC is inhibited by Gö6976 and Gö6983 (10 μM; *n* = 6 embryos each condition standardized to NGF values and tested by one-factor ANOVA and Dunnett’s *post hoc* comparison). ***E***, ***F***, PKC inhibition after 12 h rescues axons from cytoskeletal degeneration induced by 24 h of NGF deprivation. ***E***, Axoquant2.0 axon density output curves are presented with mean and SEM (*n* = 9 for all conditions except *n* = 7 for Gö6983). ***F***, Axon density was analyzed within 1000-μm bins using two-factor ANOVA and Tukey’s *post hoc* analysis. Median, min/max, and 25/75% are indicated; ***p < 0.001, ****p < 0.0001.

In several settings, NOX complexes are activated by PKC (Frey et al., 2002; Ibi et al., 2008; Cosentino-Gomes et al., 2012), and we therefore asked whether PKC activity mediates Ca^2+^ entry and developmental sensory axon degeneration. First, we performed gain of function experiments to determine if PKC activation is sufficient to drive Ca^2+^ influx by exposing axons to PMA, a potent PKC activator. Figure 4*A*,*B* shows that PMA-mediated PKC stimulation potently induces Ca^2+^ influx in axons Next, we applied PKC inhibitors to axons during NGF deprivation. Figure 4*C*,*D* shows that PKC inhibitors Gö6976 and Gö6983 each strongly blocked the Ca^2+^ influx induced by 15 h of NGF deprivation, and Figure 4*E*,*F* shows that these compounds significantly improve axon integrity observed after 24 h of NGF deprivation. These results are consistent with the hypothesis that PKC-dependent NOX activation drives TRPV1 opening following NGF deprivation.

**Figure 5.**
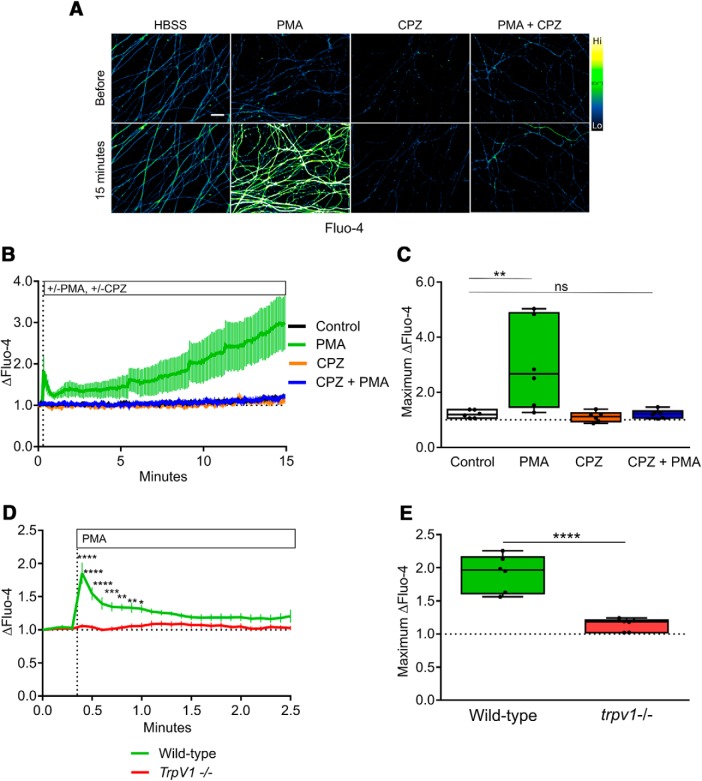
TRPV1 mediates PKC-dependent axonal Ca^2+^ flux. ***A***, Representative images of DRG axons loaded with Fluo-4 and live-imaged during stimulation with PKC activator PMA in the presence or absence of TRPV1 inhibitor CPZ (10 μM). TRPV1 inhibition abolished the axonal Ca^2+^ response to PKC activation. ***B***, Time course of the Fluo-4 responses to Ca^2+^ influx during the 15-min recording period (*n* = 6, mean and SEM are indicated). ***C***, Maximum responses during the recording period were analyzed using two-factor ANOVA and Tukey’s *post hoc* comparison; indicated are median, min/max, and 25/75%. ***D***, The axonal Ca^2+^ response to PKC activation was absent in axons of *TrpV1-*knock-out DRG (*n* = 6, mean and SEM are indicated and data were analyzed by two-factor ANOVA and Sidak’s *post hoc* comparison). ***E***, The maximum Fluo-4 responses to axonal Ca^2+^ were analyzed by an unpaired, two-tailed *t* test and indicated are median, min/max, and 25/75%; *p < 0.05, **p < 0.01, ***p < 0.001, ****p < 0.0001.

To determine whether any of this PKC-dependent Ca^2+^ influx occurred via TRPV1, axons were exposed to PMA in the absence and presence of capsazepine. Interestingly, the TRPV1 blocker capsazepine strongly reduced the PMA-induced Ca^2+^ entry into axons, suggesting that PKC mediates Ca^2+^ entry into sensory axons mainly via TRPV1 (Fig. 5*A–C*). To confirm this, we examined the effect of PMA on Ca^2+^ entry in DRG sensory axons derived from wild-type and TRPV1^-/-^ embryos; Figure 5*D*,*E* shows that the PMA-induced Ca^2+^ influx that occurs in wild-type axons is virtually absent in TRPV1^-/-^ axons. Together, these results suggest that TRPV1 is the predominant Ca^2+^ channel activated by PKC in sensory axons.

**Figure 6.**
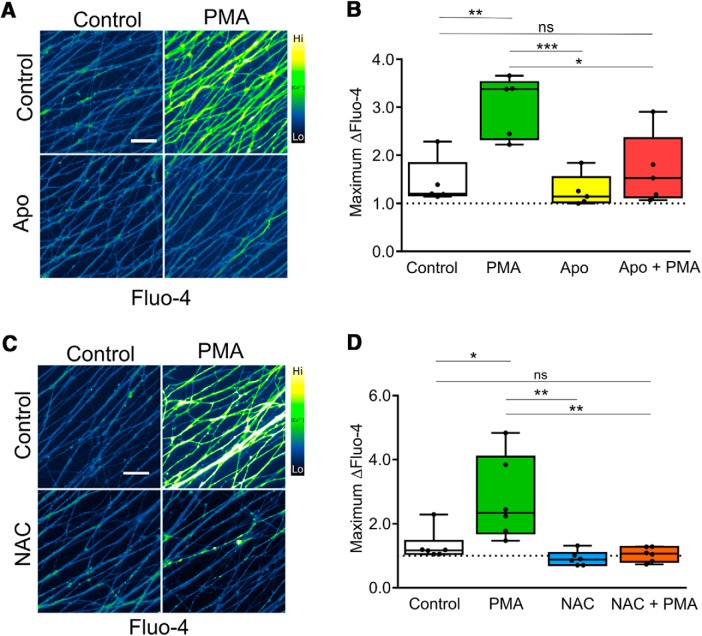
PKC-dependent Ca^2+^ flux requires ROS from NOX complexes. ***A***, Representative images of DRG axons loaded with Fluo-4 and stimulated with PMA in the presence or absence of apocynin (10 μM). ***B***, PKC-induced Ca^2+^ influx was significantly reduced by NOX complex inhibition by 10 μM apocynin (*n* = 5 pooled experiments analyzed by two-factor ANOVA and Tukey’s *post hoc* comparison). ***C***, Representative images of DRG axons loaded with Fluo-4 and stimulated with PMA in the presence or absence of NAC (20 mM). ***D***, PKC activation with PMA (100 nM) induced axonal Ca^2+^ influx that was abolished by co-application of antioxidant NAC at 20 mM (*n* = 6 pooled experiments analyzed by two-factor ANOVA and Tukey’s *post hoc* comparison). Plotted are the maximum Fluo-4 responses (relative to baseline values) during the recording period. Indicated are median, min/max, and 25/75%; *p < 0.05, **p < 0.01, ***p < 0.001.

In our final experiments, we asked whether PKC-dependent TRPV1 channel activity requires NOX activation and ROS generation. Axons were exposed to PMA in the absence and presence of apocynin (to inhibit NOX complexes) or NAC (a ROS scavenger) and assessed for Ca^2+^ entry. Figure 6 shows that both apocynin (Fig. 6*A*,*B*) and NAC (Fig. 6*C*,*D*) suppress PMA-induced Ca^2+^ entry. Taken together, our data indicate that a direct PKC > NOX > ROS signaling cascade activates TRPV1 in sensory axons and that this cascade plays a critical role in the developmental axon degeneration of DRG sensory axons.

## Discussion

Axon terminals innervating the skin can be induced to degenerate by clinical application of capsaicin, an exogenous TRPV1 agonist, which activates Ca^2+^ influx upstream of mitochondrial dysfunction and cytoskeletal degeneration, indicating that the molecular machinery is present within DRG axons to execute TRPV1-dependent neurite degeneration (Jancso et al., 1985; Gibbons et al., 2010; Chiang et al., 2015). The Ca^2+^-dependent apoptotic death of retinal ganglia cells, a neuronal subtype, subjected to experimental glaucoma *in vitro* was also found to require TRPV1, as did apoptosis of cortical neurons exposed to oxygen/glucose deprivation (Shirakawa et al., 2008; Sappington et al., 2009). We recently reported that cytoskeletal fragmentation of DRG axons is rescued by the Ca^2+^-specific chelator EGTA during NGF deprivation *in vitro,* highlighting a key role for Ca^2+^ in developmental degeneration and prompting the current study to understand its source and regulation (Johnstone et al., 2018). NGF withdrawal resulted in formation of spheroid membrane protrusions on axons characteristic of apoptotic-like decoupling of membrane from underlying cytoskeletal structural support, accompanied by elevated axoplasmic free Ca^2+^ concentration.

In the present study, we found that the rise of Ca^2+^ occurred before the appearance of membrane spheroids and degeneration of the axon, indicating a late role for Ca^2+^ in axon degeneration induced by NGF deprivation. Cultures of DRG deprived of NGF were rescued from Ca^2+^ influx by TRPV1 antagonist capsazepine, and pharmacological and genetic TRPV1 loss-of-function rescued cytoskeletal integrity. We hypothesized that ROS promote developmental degeneration upstream of TRPV1 and Ca^2+^ influx during trophic withdrawal. Consistent with this, antioxidant NAC rescued axons from Ca^2+^ influx and from degeneration, as did NOX complex and PKC inhibition. Our results reveal for the first time that a PKC > NOX complex > ROS > TRPV1 > Ca^2+^ signaling axis mediates developmental axonal degeneration in DRG neurons.

### ROS mediate developmental degeneration

We sought to place TRPV1 in a signaling context downstream of NGF withdrawal by identifying its mode of regulation. Although TRPV1 is activated by diverse stimuli including protons, heat, lipids, and plant and invertebrate defense compounds, we hypothesized that ROS are required for activation of TRPV1 in the context of developmental degeneration for several reasons: (1) intraneuronal Ca^2+^ tone and elevated oxidative status are mutually enhancing in diverse physiologic settings and in disease states (Liu et al., 2003; Mattson, 2007; Bezprozvanny, 2009; Marambaud et al., 2009; Tan et al., 2011; Deheshi et al., 2015; Schampel and Kuerten, 2017; Carrasco et al., 2018); (2) ROS are crucial for TRPV1-mediated Ca^2+^ dynamics in nociception and inflammation, two paradigms in which TRPV1 has been extensively studied (Salazar et al., 2008; Chuang and Lin, 2009; Wang and Chuang, 2011; Ding et al., 2016; Hargreaves and Ruparel, 2016; Ogawa et al., 2016); (3) cultured sympathetic neurons and PC12 cells, which like DRG both require NGF for outgrowth and survival, can be rescued from neurite degeneration and death by an antioxidant (Ferrari et al., 1995); and (4) redox regulation of TRPV1 is well established, and intracellular redox-active cysteine residues that are targeted within TRPV1 have been identified (Ibi et al., 2008; Salazar et al., 2008; Lin et al., 2015; Ding et al., 2016; Morales-Lázaro et al., 2016; Nazıroğlu, 2017). Based on these findings, we hypothesized that NGF deprivation results in ROS generation that is required for the TRPV1-mediated Ca^2+^ influx in DRG axons and our results clearly demonstrate this to be the case.

### NOX complexes generate ROS during trophic factor deprivation

What is the origin of ROS in an NGF-deprived sensory axon? NOX complexes are a family of membrane-associated oxidases that generate superoxide via electron transfer from NADPH that participate in redox regulation of downstream targets (Frey et al., 2002; Geiszt et al., 2003; Hilburger et al., 2005; Cao et al., 2009; Petry et al., 2010). In embryonic sympathetic neurons, which like DRG depend on NGF for survival, genetic deletion of NOX isoform NOX2 rescued axons from degeneration, as did pharmacological NOX complex inhibition (Tammariello et al., 2000; Hilburger et al., 2005). Further, a growing body of literature points to an intimate link between NOX complex activation and TRPV1-mediated Ca^2+^ dynamics in pain and inflammation paradigms (Ibi et al., 2008; Lin et al., 2015; Ding et al., 2016; Nazıroğlu, 2017). It is firmly established that ROS are generated as a consequence of mitochondrial dysfunction during neurite degeneration and apoptosis, and it is likely that mitochondria-derived ROS are generated as axons degenerate (Kirkland et al., 2002; Kirkland and Franklin, 2003; Celsi et al., 2009; Hajnóczky et al., 2015; Prudent et al., 2015). However, in DRG axons stimulated to degenerate with capsaicin, loss of mitochondrial membrane potential is a consequence, not a cause, of TRPV1-mediated Ca^2+^ influx (Czeh et al., 2005; Chiang et al., 2015). Since NOX complex inhibition rescued axons from Ca^2+^ influx and degeneration in our setting, we conclude that NOX complexes are the primary source of ROS that contributes to TRPV1 activation in degenerating axons.

### A PKC > NOX > ROS > TRPV1 > Ca^2+^ signaling cascade mediates Ca^2+^ influx in DRG axons

PKC-dependent phosphorylation of the core activator subunit p47^phox^ plays a critical role in the assembly and activation of several NOX complexes (El-Benna et al., 2008). Typical PKCs are activated by Ca^2+^ and diacylglycerol (DAG) and the pharmacological DAG mimetic PMA is a potent PKC activator (Grimes et al., 1996; Frey et al., 2002; Ibi et al., 2008; Cosentino-Gomes et al., 2012; Lucke-Wold et al., 2015). Our finding that *TRPV1* genetic knock-out completely ablated the axonal Ca^2+^ influx response to PMA is consistent with published *in vivo* data indicating that the noxious effect of intraplantar PMA injection was mediated by TRPV1 (Bölcskei et al., 2005). Further, our evidence for axonal signaling cassette comprised of PKC > NOX > ROS > TRPV1 > Ca^2+^ is consistent with data from other settings (e.g., inflammation) that have linked extracellular signaling to TRPV1 activation (Ibi et al., 2008; Lin et al., 2015; Ding et al., 2016; Nazıroğlu, 2017).

### How does TrkA transduce a prodegenerative signal to the PKC/NOX/TRPV1 signaling cassette?

The apical molecular events that link NGF deprivation to PKC activation remain unknown. In this regard, it is notable that whereas NGF-bound TrkA activates pro-survival signaling; together with the p75 neurotrophin receptor (p75^NTR^), recent studies have suggested that when withdrawn from ligand, TrkA can actually drive pro-degenerative pathways (Barker and Shooter, 1994; Nikoletopoulou et al., 2010; Feinberg et al., 2017). One of the signaling events activated by TrkA is the phospholipase C (PLC)γ pathway and in subsequent studies it will be interesting to determine if TrkA-dependent PLCγ activity plays a role in PKC activation after NGF withdrawal. This intriguing possibility would be consistent with results from Calissano and colleagues that show that in NGF-treated hippocampal neurons, TrkA and PLCγ phosphorylation is rapidly lost after NGF withdrawal but both rise several hours later (Matrone et al., 2009). Recent work has indicated a role for TNFR-family member death receptor 6 (DR6) in developmental degeneration downstream of NGF deprivation (Nikolaev et al., 2009); whether DR6 enhances PKC activation in this context is not yet known.

In closing, we have established a novel role for TRPV1 in developmental sensory axon degeneration and established the existence of a PKC > NOX > ROS > TRPV1 > Ca^2+^ signaling cassette in this setting. These studies have been limited to peripheral sensory axons but TRPV1 is broadly expressed in the central nervous system, and sporadic evidence has suggested a role for TRPV1 in synapse remodeling (Ramírez-Barrantes et al., 2016). Future studies should test the intriguing possibility that selective pruning of neurites is accomplished by locally-restricted TRPV1 activation, not only in the periphery but also in the brain.

## Synthesis

Reviewing Editor: Jeffery Twiss, University of South Carolina Decisions are customarily a result of the Reviewing Editor and the peer reviewers coming together and discussing their recommendations until a consensus is reached. When revisions are invited, a fact-based synthesis statement explaining their decision and outlining what is needed to prepare a revision will be listed below. The following reviewer(s) agreed to reveal their identity: NONE. Note: If this manuscript was transferred from JNeurosci and a decision was made to accept the manuscript without peer review, a brief statement to this effect will instead be what is listed below.

I have carefully reviewed the critiques from J Neurosci submission and the authors' rebuttal. The modest edits to the manuscript along with the authors' rebuttal fully address the concerns raised from both reviewers. This work gives new insight into mechansims of axon degeneration following neurotophin deprivation. The in vitro work is completely appropriate for answering the questions, and Johnstone et al. have uncovered a new role for TRPV1 in axon degeneration following NGF withdrawal. This has important implications for neural development and dying back axonopathy in neurodegeneration. I have only excessively minor points that would improve interprepration of the figures and reading:

if the authors add ‘(relative to control)’ to Y axis label in Figs 1B and D, I think it will be easier for the reader to interpret - i.e., as they use in Fig 2B.

For Figs 2B, I agree that the ‘null’ designation would be more clear if the authors used TRPV1 +/+ and -/- designation as they have used in Fig 5D and E.

Using the designations of ‘PMA + CPZ’ for Figs 5B & C (as in Fig 5A) and ‘Apo + PMA’ & ‘NAC + PMA’ for Figs 6 B & D would make the annotation easier to follow between panels.

Line 304, ‘e’ is missing in circumstances.
